# Role of Imaging in Chronic Inflammatory Demyelinating Polyneuropathy: A Systematic Review

**DOI:** 10.1111/ene.70226

**Published:** 2025-06-01

**Authors:** Stefano Tozza, Emanuele Cassano, Carmen Erra, Mario Muto, Francesco Habetswallner, Fiore Manganelli

**Affiliations:** ^1^ Department of Neuroscience, Reproductive and Odontostomatological Science University of Naples Federico II Naples Italy; ^2^ Clinical Neurophysiology Unit, Cardarelli Hospital Naples Italy; ^3^ Neuroradiology Department, Cardarelli Hospital Naples Italy

**Keywords:** CIDP, MRI, ultrasound

## Abstract

**Introduction:**

Chronic Inflammatory Demyelinating Polyneuropathy (CIDP) is a treatable immune‐mediated neuropathy with a relapsing‐remitting course and symmetrical proximal and distal weakness. Diagnosis relies on nerve conduction studies (NCS) to detect demyelination but can be difficult in atypical cases. In such instances, nerve ultrasound (US) and MRI of the brachial and lumbosacral plexuses help improve diagnostic accuracy and guide treatment. This review examines the role of imaging in CIDP, focusing on its contribution to diagnosis, prognosis, and follow‐up.

**Methods:**

A total of 183 articles were identified in the PubMed database using the search terms: “CIDP AND imaging,” “CIDP AND ULTRASOUND,” and “CIDP AND MRI.” Based on predefined inclusion criteria, 106 articles were selected for review (63 related to US and 43 to MRI). From each included study, data were extracted on the study population, imaging protocols used, outcome measures applied, and main findings relevant to the review's aim.

**Results:**

The most used ultrasound and MRI protocols, along with their associated outcome measures, are discussed. Furthermore, the roles of each imaging modality in diagnosis, prognosis, and follow‐up are analysed.

**Conclusion:**

Although NCS remain the primary instrumental test for the diagnosis of CIDP, US and MRI can be valuable adjuncts in cases with diagnostic uncertainty. Additionally, these imaging modalities may be more useful than NCS in prognostic evaluation, helping in predict treatment response and monitoring subclinical disease activity.

## Graphical abstract

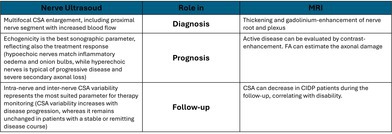

## 1 Introduction

Chronic Inflammatory Demyelinating Polyneuropathy (CIDP) is a rare and acquired disease of the Peripheral Nervous System (PNS). A correct diagnosis can be challenging since the clinical picture may be heterogeneous, and different conditions can mimic CIDP. In fact, the last EAN/PNS guideline recognized different phenotypes [1]. The typical CIDP is characterized by progressive or relapsing, symmetric, proximal and distal muscle involvement in the four limbs along with sensory deficit. On the other hand, the CIDP variants encompass the distal CIDP (distal sensory loss and muscle weakness predominantly in lower limbs), the multifocal CIDP (known as Lewis Sumner syndrome, characterized by a multifocal sensory‐motor pattern, usually asymmetric with a predominant involvement of upper limb), the focal CIDP (sensory and muscle involvement in only one limb), the motor CIDP (exclusively or predominantly motor symptoms and signs), and the sensory CIDP (exclusively or predominantly sensory symptoms and signs).

Beyond clinical features, CIDP diagnosis is established on electrophysiological findings by demonstrating nerve conduction abnormalities fulfilling demyelinating criteria according to the EAN/PNS task force (distal motor latency prolongation, nerve conduction slowing, prolongation or absence of F‐wave, motor conduction block, abnormal temporal dispersion, prolongation of compound muscle action potential duration).

Lastly, if a diagnosis of possible CIDP is made or if electrodiagnostic testing does not fulfill demyelinating features, a series of supporting criteria (response to treatment, imaging, cerebrospinal fluid, or nerve biopsy) may be applied to confirm the diagnosis.

EAN/PNS guidelines advise using nerve ultrasound (US) and/or brachial or lumbosacral plexus magnetic resonance imaging (MRI) in adult patients fulfilling diagnostic criteria for possible CIDP. Nerve US in CIDP patients demonstrated enlargement, computed as Cross‐Sectional Area (CSA), mainly of proximal nerve segments in arm nerves and spinal nerve roots [1]. Nerve US is a low‐cost, widely available, non‐invasive procedure with a moderate diagnostic accuracy in diagnosing CIDP. Beyond the most commonly used variable (CSA) to distinguish CIDP patients from healthy controls, a nerve may be considered pathological when additional ultrasound features are assessed, as discussed in this study. However, abnormal ultrasound findings can also be present in other peripheral nervous system disorders, and nerve US alone may not reliably differentiate among different diseases. The use of MRI in CIDP showed enlargement and/or increased signal intensity of the nerve root on T2‐weighted MRI sequences and/or contrast enhancement. However, the low inter‐rater reliability, lack of objective cut‐off values, and high cost of MRI contribute to defining this procedure as a supportive tool in diagnosing CIDP [1].

The aim of this review is to focus on the role of imaging in CIDP and its contribution to diagnosis, prognosis, and follow‐up by a systematic review of the literature.

## 2 Methods

### 2.1 Search Strategy

Keywords related to the topics were used to search relevant research articles in the PUBMED database. Only articles including human data, peer‐reviewed and published in English from 2000 to 6th March 2024, were included. The following search terms were used: “CIDP AND imaging”, “CIDP AND ULTRASOUND”, “CIDP AND MRI”. Eligible studies were identified by abstract and full‐text screening, according to predefined criteria. Inclusion criteria were: – article relevant and focused on topics of this review, including the role of imaging (US, MRI) in the diagnosis, prognosis, and follow‐up of CIDP; − adult (> 18 years) population; − the following study type: multicentre study, observational study, case series. Exclusion criteria were: – not relevant and focus on topics outlined in the inclusion criteria; − not in adult (< 18 years) population; − not the following study types: guideline, meta‐analysis, review, case report, letters, commentaries, or editorials. The following information from the included articles was extracted: study population, used imaging protocol, used outcome measure, and main results concerning the review focus. All included publications were assessed to ensure that there was no duplicate reporting of data.

## 3 Results

**FIGURE 1:**
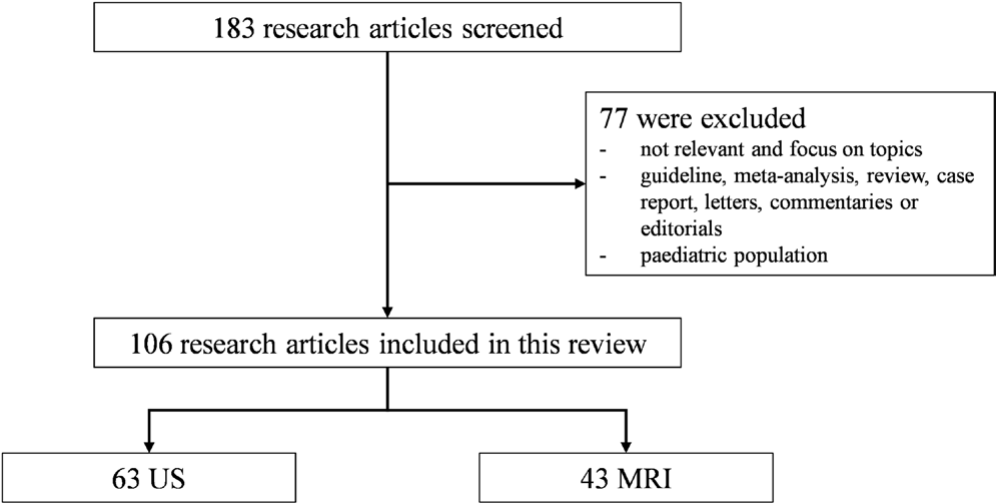
Flowchart of the article selection process in this systematic review.

A total of 183 papers were identified from the PUBMED database. After abstract and full‐text screening, 106 articles were assessed. Included articles were categorized by imaging type (63 research of US and 43 research of MRI) (Figure 1).

### 3.1 Nerve Ultrasound

In Table S1 The research articles regarding nerve US were summarized. Nerve US lets us visualize the nerve along all segments up to their origin. Normal nerve appearance in the axial plane is characterized by a typical “honeycomb” feature with hypoechoic individual fascicles surrounding hyperechogenic epineurium [2]. Different sonographic features can be noticed in CIDP patients, including changes in nerve size (CSA), enlargement distribution (homogeneity), echointensity, fascicle dimensions, and Doppler signal.

#### 3.1.1 Nerve US Protocol

In the literature, different nerve US protocols are present, as well as several US scores have been described. A linear probe, with US frequency ranging from 5 to 18 MHz, is typically used, and higher frequencies are applied for more superficial nerves, while lower frequencies are used for deeper nerves. Less frequently, Ultra‐High Frequency US (UHFUS 30–70 MHz) has been performed, obtaining more detailed information on the changes in the nerve structure [3]. A complete nerve US protocol should include bilaterally the vagus nerve (in carotid sheath), median nerve (at elbow, forearm and the wrist), ulnar nerve (at Guyon's canal, ulnar sulcus and forearm), radial nerve (at the spiral groove and the Frohse arcade) tibial nerve (at popliteal fossa and ankle), fibular nerve (at the popliteal fossa) and sural nerve (at calf), brachial plexus, and the diameter of the 5th and the 6th cervical spinal roots (at leaving the intervertebral foramen and the processus transversus, with transverse scan for assessing nerve morphology such as CSA, fascicles, echogenicity and longitudinal views for evaluating nerve course and identifying potential compression sites) [4]. However, US studies generally investigated only part of the complete protocol, and different scores were applied. A short protocol, including the median nerve (at the forearm and upper arm) and the C5 cervical root, demonstrated a higher sensitivity with respect to the EFNS/PNS 2010 criteria in those patients who display normal NCS [5, 6].

#### 3.1.2 Outcome Measures

##### 3.1.2.1 Cross‐Sectional Area (CSA)

The CSA has been widely used as a main outcome measure in CIDP patients. CSA was measured in nerve axial view by tracing inside the hyperechoic rim of the nerve epineurium. The increase of CSA quantifies the enlargement present in CIDP patients. Enlargement was defined on published cut‐off values or by comparison with other diseases. Accordingly, based on CSA measures, different scores were developed to assess PNS involvement.

The most used score is the US pattern sum score (UPSS), which assesses the ability to distinguish between acute and subacute onset polyneuropathies, whether axonal or demyelinating [7]. The total score, ranging from 0 to 20 points, was computed based on three sub scores: CSA values of peripheral sensorimotor nerves (UPSA, 0–16 points), the cervical spinal nerves C5, C6, and the vagal nerve (UPSB, 0–3 points) and the sural nerve (UPSC, 0–1 point). The UPSA explores the peripheral nerve enlargement in the median nerve at the mid arm, elbow, and forearm; ulnar nerve at the mid arm and forearm; tibial nerve at the popliteal fossa and ankle; and fibular nerve at the lateral popliteal fossa. For each nerve segment, 2 points are assigned when enlargement is > 150% and 1 point when the CSA value is between 100% and 150% of the defined maximal values. The UPSB evaluates the proximal involvement of any neuropathy through the study of the cervical spinal nerves 5 and 6 and the vagal nerve; each enlargement is rated with 1 point. Lastly, the UPSC evaluates the sural nerve as a purely sensory nerve, assigning only 1 point. The UPSS and subscores have a sensitivity > 85% with a general PPV of 80%–87.5% for the diagnosis of CIDP (UPSA ≥ 7 or UPSS ≥ 10 points), GBS (UPSB alone ≥ 1), vasculitis neuropathy (intermediate UPSS 3–9 points), or axonal neuropathy (UPSS < 3 points) [8].

Another used score is the Bochum US score (BUS), which was developed to differentiate between CIDP and AIDP [9]. BUS is computed by measuring the ulnar nerve CSA at the upper arm and the Guyon channel, the radial nerve CSA at the spiral groove, and the CSA sural nerve at the calf. Each nerve enlargement (according to boundary values) is scored with 1 point, with a maximum BUS of 4 points. A total score > 2 points endorses CIDP diagnosis with a sensitivity of 87.5% [10] and a score < 2 points supports GBS with a sensitivity of 84.2% [11]. BUS was used as a first‐phase analysis of a step‐by‐step protocol (neuritis US protocol—NUP) [12]. When BUS was < 2 points, the authors suggested exploring the median and the ulnar nerves at the mid‐forearm or the tibial nerve at the ankle. If the enlargement is found in at least one segment, a diagnosis of Multifocal Motor Neuropathy can be made. If no CSA increase was found, a third step was required to analyze the median nerve at the carpal tunnel or the ulnar nerve at the elbow to support the diagnosis of multifocal CIDP. If no pathological changes were detected at each step, US findings could be suggestive of vasculitis or paraproteinemic PNP. NUP revealed a sensitivity for BUS ≥ 2 for CIDP of > 80% with a PPV > 80%, while Step 2 showed a sensitivity of 100% for MMN with a PPV of > 50%, and Step 3 showed a sensitivity of 100% with a PPV of 50% for MADSAM [12].

##### 3.1.2.2 Enlargement Distribution

Nerve enlargement distribution is useful to discriminate hereditary (Charcot–Marie‐Tooth type 1—CMT1) vs. acquired demyelinating neuropathy. A study, by evaluating the ulnar and the median nerves at the upper arm and the forearm, recognized four different patterns [13]. Pattern 1: no nerve enlargement at all (none); pattern 2: slight enlargement < twice the median normal (mild); pattern 3: normal next to an enlarged segment > twice the median normal (regional); pattern 4: all segments enlarged, at least one segment > twice (diffuse). Pattern 4 was found in about 90% of CMT1 patients, with a positive predictive value (PPV) for Pattern 4 in CMT1 ~60%, while patterns 1–3 were heterogeneously distributed among the acquired neuropathies.

By using the UPSS, the homogeneity score (HS) was developed to improve the sensitivity of nerve US for differentiating between CIDP and CMT [14, 15]. The homogeneity score is rated as none (no enlargement at all), regional (normal nerve segments next to enlarged), inhomogeneous diffuse (enlarged segments > 100% and < 150% of cut‐off values; scored with 1 point), homogeneously 1° (all segments > 100% and < 150%; scored with 2 points) and homogeneously 2° (all segments > 150%; scored with 3 points). The tibial, the ulnar, and the median nerves are evaluated with a total score ranging from 0 to 9 points. In CMT1 patients, the overall UPSS and the homogeneity score were significantly higher than in acquired neuropathy [8, 14, 15].

The enlargement distribution can also be easily evaluated by computing the intra‐nerve (within the same nerve) CSA variability (= maximal CSA/minimal CSA) and inter‐nerve (between different nerves) CSA variability (= maximal intra‐nerve variability/minimal intra‐nerve variability) [16]. Intra‐nerve CSA variability was more often observed in CIDP than in CMT [17], whereas inter‐nerve CSA variability was more often seen in asymmetric pathologies, such as MADSAM and MMN [18].

##### 3.1.2.3 Nerve Echogenicity

Echogenicity of peripheral nerves provides insight into the structural damage of peripheral nerves in polyneuropathies, since echointensity is typically reduced in nerve injury and is usually associated with loss of the normal fascicular architecture described above. Different methods for measuring nerve echogenicity have been used up to now: the measurement of the mean grey value [19], of the fraction of black [20], as well as qualitative patterns [21].

Padua and colleagues described pattern classes by adding to the nerve enlargement the echogenicity of the nerve [22]. Authors have recognized 3 pattern classes in CIDP patients: class 1 shows enlarged, hypoechoic nerves; class 2 shows hypo‐ and hyperechoic nerve fascicles; class 3 shows not enlarged fascicles with hyperechoic texture. Most CIDP patients showed class 1. If class 3 occurred more often in chronic cases, correlating with disease duration, class 1 was more often found in early disease stages. Thus, hypoechoic enlarged fascicles may be an expression of nerve edema and acute inflammation, while hyperechoic enlargement could be an expression of fibrosis, onion bulb formation, and chronic remyelination [8, 22].

##### 3.1.2.4 Fascicle Dimension

Frequently, not only can the enlarged CSA of the whole nerve be observed, but also single enlarged fascicles in CIDP patients. The selective enlargement of individual fascicles, together with the fading fascicular structure, was the most frequent finding in CIDP patients [20].

##### 3.1.2.5 Doppler Signal

The Doppler effect is a change in US frequency reflected from an object, such as a red blood cell, moving toward or away from the transducer. This can be used to demonstrate changes in the vascularity of peripheral nerves and surrounding structures. Normal nerve does not have any detectable blood flow. Vice versa, the presence of Doppler flow is abnormal in peripheral nerves and indicates hypervascularity, which has been described in inflammatory neuropathies such as CIDP. Increased nerve vascularization was considered a possible marker for disease activity in CIDP patients [23, 24]. However, hypervascularity has also been described in other conditions such as compressive neuropathy, diabetic polyneuropathy, infective neuropathy (e.g., leprosy), POEMS, peripheral nerve vasculitis, and tumor nerve [25].

#### 3.1.3 Roles of Nerve US

##### 3.1.3.1 Diagnosis

Although a direct correlation between morphological sonographic changes and electroneurography has not been definitely established, US technique demonstrated to be able to discriminate between CIDP and healthy control (HC) and also among different PNS diseases, including acute neuropathy as GBS, hereditary demyelinating neuropathy as CMT1, axonal neuropathy (idiopathic, ALS, hereditary TTR amyloidosis), paraproteinemic neuropathy as anti‐MAG neuropathy and POEMS syndrome, dysmetabolic as diabetic demyelinating neuropathy and other dysimmune neuropathies as Multifocal Motor Neuropathy (MMN).

US features in CIDP patients are characterized by multifocal enlargement of nerves in non‐compression sites, individual enlarged fascicles, hypoechoic nerves at early stages, and hyperechoic signal as a sign of axonal damage and fibrosis [26]. Notably, sonographic enlargement of proximal nerve segments and roots represents the key feature of CIDP [27] and can explain the CSF protein elevation [28]. Moreover, patients with CIDP also show an increase in blood flow as occurs in other forms of inflammatory neuropathies (e.g., GBS) [23, 29].

The evaluation of vagus nerve CSA allows for discrimination between CIDP and other neuropathies. In one study, CMT1A patients displayed, over a diffuse and homogeneous nerve enlargement, a vagus nerve CSA increase greater than that of CIDP, and a cut‐off value of 1.5 mm^2^ produced a sensitivity of 79.3% and a specificity of 91.3% in the diagnosis of CIDP [30]. Moreover, the vagus nerve enlargement, together with sparing of the sural nerve, allowed for discrimination between GBS and CIDP in the early stage [29].

US evaluation might also be able to discriminate between the variants of CIDP. A single study focused on the distribution pattern of nerve enlargement in clinical subtypes showed that diffusely moderate enlargement was more common in typical CIDP and multifocal CIDP, while proximal regional enlargement was more common in distal and pure motor CIDP [31].

Lastly, USs may be able to show typical sonographic changes also in CIDP patients in which NCS do not fulfill demyelinating features according to the EFNS/PNS 2010 criteria [1]. A US study led to the recognition as chronic inflammatory neuropathy 20%–25% of patients presented with normal NCS. A short sonographic protocol including the median nerve at the forearm (> 10 mm^2^) and at the upper arm (> 13 mm^2^), and the C5 cervical root (> 8 mm^2^) had higher sensitivity (97.4% vs 78.9%) but lower specificity (69.4% vs 93.5%) than EFNS/PNS 2010 criteria [5, 6].

##### 3.1.3.2 Prognosis

In the last few years, researchers focused their attention on the prognostic role of nerve US in CIDP. Nerve enlargement distribution can help in distinguishing patients with chronic CIDP (who show a more generalized distribution pattern) from CIDP patients at onset [14]. Furthermore, a BUS > 4 may identify patients with disease progression (sensitivity 80% and specificity 88%) [24]. However, the best sonographic feature that can give information for prognosis is the nerve echogenicity [32]. Hypoechoic enlarged nerves and fascicles reflect inflammatory edema and onion bulbs, while patients with progressive disease and severe secondary axonal loss present small hyperechoic nerves, reflecting a scar‐fibrous remodeling [20, 22]. The nerve echogenicity may also predict the treatment response: patients with hypoechoic enlarged nerves experienced a significantly improved clinical course during treatment than those with increased echointensity associated with axonal damage, difficult to treat with current anti‐inflammatory therapies [21]. Moreover, the degree and distribution of nerve enlargement may suggest which type of immunomodulatory treatment patients deserve. Patients with moderate enlargement and those with proximal regional enlargement showed a higher response to steroids than to IVIg [33, 34]. Lastly, the decrease of CSA after treatment may also indicate a better prognosis [31].

##### 3.1.3.3 Follow‐Up

Although nerve echogenicity can be considered during the follow‐up period in establishing the disease activity [22], the nerve enlargement variability (intranerve and internerve CSA) represents the most suited parameter for therapy monitoring. In fact, CSA variability increases with disease progression, whereas it remains unchanged in patients with a stable or remitting disease course [35, 36].

### 3.2 MRI in CIDP

In Table S2 The research articles regarding nerve MRI. MRI is another modality for visualising the nerve changes in CIDP patients. Roots, plexus, and peripheral nerve MRI can be a valuable addition in the diagnostic work‐up, as proximal nerve segments can be easily assessed, while NCS cannot study such regions appropriately [37]. The hallmark is thickening of peripheral nerves, plexus, and nerve roots, which can appear hyperintense in T2 sequences and can show gadolinium enhancement.

However, different radiologists may provide different qualitative results for the same image due to low inter‐rater reliability, resulting in a limited diagnostic accuracy of qualitative assessment of MRI in CIDP [38, 39]. Therefore, apart from qualitative nerve changes and alterations of the conventional MRI protocol, promising new MRI methods that are capable of measuring nerve hypertrophy, hyperintensity, and gadolinium enhancement through quantitative parameters have emerged.

#### 3.2.1 MRI Protocol

The standard MRI protocol for CIDP typically involves the use of specific sequences and techniques for assessing nerve roots and brachial and lumbosacral plexus. The conventional sequences include the Short Tau Inversion Recovery (STIR) that evaluates the signal intensity, useful for detecting hypertrophy and swelling of nerve structures; the T2‐weighted imaging that assesses the size and signal intensity and evaluates the extent of nerve damage and inflammation; the Diffusion Tensor Imaging (DTI) that assesses the integrity of nerve fibres and the degree of demyelination, providing information on the direction and magnitude of water diffusion within the nerve tissue; and the gadolinium‐enhanced MRI that evaluates the presence of inflammation and oedema, identifying areas of active inflammation and demyelination.

Subgroup analysis for the MRI sites (brachial plexus and lumbosacral plexus) showed higher sensitivity (75%) and specificity (89%) for the lumbosacral plexus with respect to brachial plexus (60% and 82%, respectively), suggesting that the lumbosacral plexus may be more likely affected in patients with CIDP [40].

#### 3.2.2 Outcome Measures

##### 3.2.2.1 Nerve Thickening

One of the main parameters evaluated by MRI is the thickening of nerve structure. Although not specific for CIDP, since it may be seen in other relatively frequent diseases, nerve hypertrophy was found in 37%–100% of CIDP cases [37, 41]. The CSA, as a two‐dimensional parameter, can be easily measured and provides more information than the one‐dimensional parameter diameter, since the nerve roots are not always circular. Wu et al. found that increased CSA correlated positively with central latency and negatively with nerve conduction velocity (NCV) [42]. Lastly, only one study investigated the volume parameter, a 3D measurement of nerve hypertrophy [43]. Overall, among these quantitative parameters, the best ratio between sensitivity and specificity was achieved by CSA (76% and 95%, respectively), while diameter had a lower sensitivity (67%) and volume a relatively low specificity (82%) [40].

##### 3.2.2.2 Nerve Hyperintensity

Nerve roots may appear hyperintense in the T2 sequence in 56%–100% of CIDP cases [37], expressing the changes in nerve architecture. Among the quantitative parameters of nerve hyperintensity, Fractional Anisotropy (FA) represents the most promising measurement in quantifying nerve intensity. FA is a valuable measure calculated on the DTI sequence that provides insights into the microstructural integrity of white matter tracts. FA is typically decreased in CIDP patients, primarily due to an increase in radial diffusivity (RD), reflecting myelin sheath damage. It is consistent with the current hypothesis that an increased RD may serve as a specific biomarker of demyelinating neuropathy [44]. Kronlage et al. found that decreased FA values were also strongly correlated with decreased NCV and increased F‐wave delay and demonstrated that an optimal FA cutoff of 0.47 yielded a sensitivity of 0.83 and a specificity of 0.94 for distinguishing CIDP patients from controls [45]. If FA yielded the highest pooled sensitivity (85%) and the highest specificity (92%), other parameters, including contrast ratio (CR), signal‐to‐noise ratio (SNR) and contrast‐to‐noise ratio (CNR), that are also associated with nerve hyperintensity, have shown a poor diagnostic value [40].

##### 3.2.2.3 Gadolinium Enhancement

Involved nerves have also been shown to exhibit enhancement on gadolinium contrast studies in 0%–69% [37]. The contrast enhancement is likely due to increased permeability of the blood‐nerve barrier, which allows contrast agents to diffuse into the peripheral root. The increased permeability is secondary to an inflammatory process involving proximal nerve segments, as occurs in CIDP. Gadolinium enhancement may indicate an active disease [46] and it may disappear during remission induced by immune therapies [47]. Quantitative parameter able to measure gadolinium enhancement is Contrast‐Enhancement Ratio (CER), defined as the ratio of contrast enhancement nerve SNR to non‐contrast enhancement nerve SNR. Su et al. demonstrated that the lumbosacral plexus showed both higher sensitivity (73%) and specificity (96%) than the brachial plexus [48].

#### 3.2.3 Roles of MRI

##### 3.2.3.1 Diagnosis

Nerve root and plexus MRI are able to discriminate between CIDP and healthy control (HC) and among other peripheral nervous system (PNS) diseases, including hereditary demyelinating neuropathy as CMT1A, motor neuron disease, and Multifocal Motor Neuropathy (MMN). Controversial instead is the ability to discriminate with axonal neuropathy, since neither diameter [49] nor did FA parameters differ between the two groups, but only the CSA [50].

An MRI study might also be able to discriminate the variants of CIDP. Focal/multifocal CIDP, with respect to the typical CIDP, displayed an asymmetric fusiform nerve hypertrophy [51, 52] with lower nerve thickening, as occurs also in distal CIDP [53]. Furthermore, sensory CIDP shows a greater thickening of dorsal roots compared to patients with a sensory‐motor phenotype, while the motor CIDP displays a thicker ventral root.

Lastly, MRI might be able to show typical changes (thickening and gadolinium‐enhancement) also in clinical CIDP patients not fulfilling demyelinating criteria at NCS, with abnormalities more asymmetrical and less diffuse [54, 55].

##### 3.2.3.2 Prognosis

An MRI study can be useful in evaluating active disease by contrast‐enhancement [46, 47] and nerve hypertrophy of the lumbo‐sacral plexus inversely correlated with changes in the clinical scale and might predict a benign course or a better therapy response [56], although other studies demonstrated that MRI is not able to predict therapeutic response [52, 57]. Moreover, MRI can predict the natural history of focal CIDP since the plexus neuropathies developed a long‐term, focal, and benign course, while the monomelic sensory‐motor and motor involvement of peripheral nerves were more likely to evolve to multifocal CIDP or MMN phenotype [58].

##### 3.2.3.3 Follow‐Up

Recently, Preisner et al. in a longitudinal study of 6 years demonstrated that nerve CSA decreased in CIDP patients, correlating with changes in disability score, including INCAT and ODSS, while FA showed correlation with electrodiagnostic testing both cross‐sectionally and longitudinally [56]. Moreover, FA may be useful in estimating axonal damage in CIDP patients [59].

## 4 Conclusions

CIDP diagnosis is often challenging because of the heterogeneous clinical presentation, and NCS abnormalities can be misinterpreted. In fact, none of the demyelinating findings are specific for CIDP, and not all patients exhibit NCS abnormalities fulfilling demyelinating criteria. The electrodiagnostic criteria can be supplemented with additional diagnostic tests, including nerve US and nerve root and plexus MRI. Although the evidence for each of these diagnostic tests is limited, since the studies are often small without the use of a clinically relevant control group, they can help clinicians in doubtful CIDP cases.

In detail, CSA measurements obtained by ultrasound and MRI show a significant correlation, although MRI consistently reports higher CSA values across all nerves. The best concordance is observed in upper extremity nerves, while nerves with larger CSA or those that are less accessible due to anatomical factors (e.g., the tibial nerve) exhibit greater discrepancies, with differences reaching up to 2–3 mm^2^ [60]. The lumbosacral plexus can be evaluated exclusively by MRI, whereas both modalities are comparable for brachial plexus assessment [60]. Notably, ultrasound offers several advantages due to its bedside applicability, cost‐effectiveness, and ease of use by neuromuscular specialists, making it a more practical and accessible tool in clinical practice.

In conclusion, we believe that nerve conduction studies (NCS) remain the primary instrumental test for the diagnosis of CIDP. However, in cases that do not fully meet the EAN/PNS criteria, ultrasound and MRI can support clinicians in refining the diagnosis. Conversely, ultrasound and MRI may be more useful than NCS for prognostic evaluation, predicting treatment response, and monitoring subclinical disease activity.

## Author Contributions

**Stefano Tozza:** conceptualization, methodology, data curation, formal analysis, writing – original draft. **Emanuele Cassano:** methodology, data curation, writing – original draft, formal analysis. **Carmen Erra:** writing – review and editing, data curation. **Mario Muto:** supervision. **Francesco Habetswallner:** supervision, writing – review and editing. **Fiore Manganelli:** writing – review and editing, supervision, conceptualization.

## Conflicts of Interest

The authors declare no conflicts of interest.

## Supporting information


**Table S1.** Summary of nerve ultrasound research articles included in the systematic review.

**Table S2.** Summary of nerve MRI research articles included in the systematic review.

## Data Availability

The authors have nothing to report.
