# Supplementary material for: Role of Imaging in Chronic Inflammatory Demyelinating Polyneuropathy: A Systematic Review
Source: Eur J Neurol. 2025 Jun 1;32(6):e70226. doi: 10.1111/ene.70226 (PMC12127570; doi:10.1111/ene.70226)
Supplement: Supplementary file 1 — Table S1. Summary of nerve ultrasound research articles included in the systematic review. [file ENE-32-e70226-s001.docx]

**Supplementary Table 1: Summary of nerve ultrasound research articles included in the systematic review**

| Reference | Years | Article | Study population | Aim and protocol | Main Finding |
| --- | --- | --- | --- | --- | --- |
| 3 | 2004 | Matsuoka | 13 CIDP *vs* 35 HC | Enlargement of cervical nerve roots | Hypertrophy of the cervical nerve roots in 69% of CIDP patients. The degree of hypertrophy was significantly associated with the level of CSF protein. |
| 4 | 2009 | Zaidman | 35 CMT *vs* 55 CIDP *vs* 21 GBS *vs* 17 MMN *vs* 90 HC | Analysis of patterns of CSA enlargement in median and ulnar nerves. Three patterns were defined based on a combination of the amount and anatomical extent of nerve enlargement: mild (type 1), regional (type 2), and diffuse (type 3). | Nerve enlargement was commonly diffuse (89%) and generally more than twice normal size in CMT1, but not in acquired disorders which mostly had either no, mild or regional nerve enlargement [CIDP (64%), GBS (95%), and MMN (100%)]. In CIDP, subjects treated within 3 months of disease onset had less nerve enlargement than those treated later. |
| 5 | 2012 | Rajabally | 14 CIDP *vs* 14 patients with sensory axonal neuropathy | Nerve width (medial to lateral diameter), thickness (anterior to posterior diameter) and CSA of median nerves at wrist and forearm. | Largest thickness in patients with CIDP. Nerves were wider and had larger CSA, but were not thicker, at wrist compared to forearm in both patient groups. |
| 6 | 2013 | Sugimoto | 10 CMT1 *vs* 16 CIDP | CSA of median, ulnar and the diameters of the bilateral fifth (C5) and sixth (C6) cervical nerve roots were measured. | In patients with CMT1, the nerve sizes were significantly larger at all evaluation sites along the peripheral nerves than in patients with CIDP, with the exception of cervical nerve roots. |
| 7 | 2013 | Zaidman | 25 CMT1 *vs* 55 CIDP *vs* 21 GBS *vs* 17 MMN | CSA of median and ulnar nerves. Degree of nerve enlargement was computed as of regions affected: none, no enlargement; mild (nerves enlarged < twice normal); regional (nerves normal in at least one region and enlarged > twice normal in at least one region) and diffuse (nerves enlarged at all four regions with at least one region > twice normal size). | Patterns of diffuse nerve enlargement can be used to screen patients suspected of having CMT1. Normal, mildly, or regionally enlarged nerves in demyelinating polyneuropathy suggests an acquired aetiology. |
| 8 | 2014 | Goedee | 11 CIDP | CSA of median, ulnar, fibular and posterior tibial nerves bilaterally. Screening for blood flow was done as well. | Multifocal nerve enlargement was present in the majority of CIDP patients. An increased vascularization of the nerve was seen in 6 of the 11 patients. Nerves with enlargement was related with a lower Sonographic nerve enlargement in CIDP is related with clinical severity. Increased nerve vascularization, a possible marker for diseases activity, can be detected in CIDP, especially in enlarged nerves. |
| 9 | 2014 | Jang | 10 CIDP *vs* 18 HC | CSA of the vagus, brachial plexus, musculocutaneous, median, ulnar, radial, sciatic, tibial, common peroneal and sural nerves. Correlations between CSAs and various clinical and electrophysiological features. | CSAs were significantly larger in CIDP patients than controls, especially at proximal and non-entrapment sites. CSAs were significantly correlated with muscle strength at initial presentation. |
| 10 | 2014 | Kerasnoudis | 35 CIDP vs 45 AIDP | definition of BUS (see text). | The BUS showed a sensitivity of 90% and specificity of 90.4% (PPV = 81.8%; NPV = 95%) in distinguishing CIDP from AIDP, when they showed  no differences in disease duration. In addition, the BUS distinguished subacute-CIDP from  AIDP with a sensitivity of 80%, specificity of 100% (PPV = 100%, NPV = 75%). |
| 11 | 2014 | Padua | 34 CIDP | Definition of Padua classification by evaluating CSA and Echogenicity (see text). | Definition of three classes of US nerve changes: large nerves with hypoechoic nerves/fascicles (class 1); large nerves with heterogeneous hypo- and hyperechoic fascicles (class 2); normal size nerve but abnormal hyperechoic array (class 3). The three classes significantly correlated with disease duration, but not with age or disability. These results offer the possibility of exploring the use of US to assess CIDP disease activity and treatment. |
| 12 | 2014 | Zaidman | 23 CIDP | The Nerve Size Index (NSI), with size measurements corrected for height, was calculated. | In remitted CIDP patients, enlarged nerves normalized or decreased. |
| 13 | 2015 | Di Pasquale | 19 CIDP *vs* 19 HC | CSA of 14 nerve segment (median, ulnar, and peroneal nerves). | The CSA of all examined nerves was significantly higher in patients than controls, more frequently at proximal level (axilla and arm). Nerve segments with abnormal US characteristics belonged to patients with a significantly longer disease duration, lower MRC sum score, and higher INCAT score; demyelinating features was inversely correlated with the CSA. |
| 14 | 2015 | Grimm | 85 patients with acute or subacute onset polyneuropathies both axonal and demyelinating | Definition of UPSS (see text). | UPS-A and UPSS operationalize to diagnose acute and subacute-onset CIDP and its variants with high sensitivity, specificity, and PPV. An increased UPS-B with normal UPSS and other sub scores may point to the diagnosis of GBS with high PPV and enables the differentiation from CIDP. |
| 15 | 2015a | Kerasnoudis | 15 HC *vs* 11 CIDP (patients with improvement, deterioration, and stable MRC Scale Score) | The CSA of median, ulnar, fibular and tibial nerves. The “intra-nerve CSA variability” (defined as maximal CSA/minimal CSA) was also considered. | CSA variability seems to be a useful method in monitoring CIDP patients. In subject with clinical improvement an increase of the CSA could be documented (hypertrophic nerve remodelling), although almost in all cases the intra-nerve CSA variability showed a reduction. |
| 16 | 2015b | Kerasnoudis | 75 HC *vs* 48 CIDP | CSA of median nerve, ulnar nerve, radial nerve, tibial nerve, fibular nerve and sural nerve. The brachial plexus was also assessed. | Higher cross-sectional area (CSA) values of the median, ulnar, radial, tibial and fibular nerve in most of the anatomic sites and brachial plexus when compared to controls. CIDP seems to show inhomogeneous CSA enlargement in brachial plexus and peripheral nerves. |
| 17 | 2015c | Kerasnoudis | 5 CIDP *vs* 6 MMN *vs* 2 MADSAM | Evaluation of the feasibility of BUS. | The cut-off value of ≥2 points in the BUS showed a sensitivity of 80% and specificity of 87.5% (PPV = 80%, NPV = 87.5%) in distinguishing CIDP from MMN or MADSAM |
| 18 | 2015d | Kerasnoudis | 35 retrospectively patients with subacute neuropathies + 10 prospective patients with subacute neuropathy | Application of BUS and its role in distinguishing acute-onset chronic from acute inflammatory demyelinating polyneuropathy | A sum score of ≥2 points in BUS and the presence of sensory symptoms were significantly more frequent in the subacute CIDP group than in the AIDP group.  BUS is a useful diagnostic tool for distinguishing subacute CIDP from AIDP. |
| 19 | 2015 | Pitarokoili | 9 CIDP | CSA of the lower limb nerves (tibial, fibular and sural nerves) | Patients with higher disability showed isolated enlarged fascicles and increased CSA of the peripheral nerves, whereas two of them showed atrophic fascicles. |
| 20 | 2016 | Athanasopoulou | 10 CIDP *vs* 8 MAG-neuropathy *vs* 11 myeloma *vs* 10 MGUS *vs* 27 HC | CSA of vagus nerve, median, ulnar, tibial, fibular and sural nerves. Moreover, the diameter of the 5th and the 6th cervical spinal nerve was measured. UPSS was computed. | In CIDP, the median values were significantly increased compared to the most groups in all measurement points except at the entrapment sites.  UPSS was higher in the CIDP group compared to the other groups, with an established cut-off to differentiate CIDP from MAG-neuropathy (UPSA 4.5; UPSB 1.5; UPSS 6.5). Hyperechoic fascicles were found more often in CIDP than in anti-MAG with predominance in the roots. |
| 21 | 2016a | Grimm | 13 CMT1 *vs* 27 CIDP *vs* 10 MADSAM *vs* 12 MMN *vs* 23 HC | CSA of median, ulnar, peroneal, tibial, sural, vagus nerve. Moreover, the diameter of cervical spinal nerve 5 and 6 were examined. UPSS and homogeneity score were also computed. Regional nerve enlargement was evaluated. | Significant enlargement was shown in all neuropathies compared to the controls, although the amount of enlargement (UPSS) was most prominent in CMT.  Homogeneous enlargement was significantly more often seen in CMT, while in CIDP the enlargement was regional, homogeneous or inhomogeneous with equal contribution. In MMN and MADSAM regional enlargement next to normal segments predominated. |
| 22 | 2016b | Grimm | 21 onset-CIDP *vs* 21 chronic-CIDP *vs* 21 HC | Application of UPSS in CIDP patients at disease onset vs patients with chronic disease. Definition of the homogeneity score and the modified UPSS (see text). | The median ultrasound values were significantly increased at the most measurement points in onset- and in chronic-CIDP compared to the control group with an accentuation in chronic-CIDP patients, which showed a more generalized pattern. |
| 23 | 2016 | Kerasnoudis | 38 CIDP *vs* 22 MMN *vs* 25 MADSAM *vs* 25 other neuropathies | Definition of NUP (see text). | The NUP led overall to correct classification in 85.7% with CIDP, 86.9% MMN and 100% MADSAM. The NUP had > 80% sensitivity and specificity in distinguishing CIDP, MMN, and MADSAM. |
| 24 | 2016 | Merola | 22 CIDP *vs* 10 MMN *vs* 70 HC | CSA of median, ulnar, peroneal, tibial, and sural nerves. Intra-nerve CSA variability (ratio between CSA max/CSA min) and side-to-side intra-nerve variability (“side-to-side” ratio of the intra-nerve CSA variability). Moreover, qualitative analysis of nerve fascicles was performed (nerves were classified as abnormal if ≥ 3 fascicles showed a fascicle cross-sectional area ≥ 2 mm^2^). | CIDP and MMN showed differential US aspects, with greater side-to-side intra-nerve variability in MMN and higher cross-sectional areas in CIDP. |
| 25 | 2017 | Carandang | 15 inflammatory neuropathy (6 CIDP, 5 GBS and 4 MMN) *vs* 16 other neuropathies (10 ALS and 6 CMT1A) *vs* 23 HC | Maximal intraneural blood flow was assessed. | INFV was significantly higher in patients with inflammatory neuropathy, with higher INFV in GBS, followed by CIDP. MMN had lower INFV. |
| 26 | 2017a | Goedee | 75 chronic inflammatory neuropathy (CIDP, MADSAM, MMN) *vs* 70 disease control (axonal neuropathy) | CSA, fascicle size, echogenicity and  vascularization were analysed in median, ulnar, fibular and posterior tibial nerves bilaterally. Brachial plexus was also studied. | Sonographic enlargement of proximal median nerve segments in the arms and brachial plexus is a key feature of chronic inflammatory neuropathies, which helps to reliably distinguish them from axonal neuropathies. |
| 27 | 2017b | Goedee | 23 CIDP *vs* 28 MMN | CSA of any trunk (superior, median or inferior) of brachial plexus.  Moreover, enlargement, T2 hyperintensity and gadolinium enhancement at brachial plexus MRI was also studied. | Abnormal findings were found more frequently in CIDP patients than MMN. Normal findings were found in 26% CIDP patients at brachial plexus MRI and in 22% at nerve ultrasound. The combination of both diagnostic tools showed normal findings only in 13%, with an increment of diagnostic performance to 83%. |
| 28 | 2017 | Grimm | 327 patients including CIDP and other neuropathies (GBS, MADSAM, MMN, vasculitis, axonal polyneuropathy, CMT1, hereditary neuropathy, anti-MAG neuropathy, amyloidosis) and HC | Comparative analysis between Padua classification *vs* BUS/NUP *vs* UPSS. | For all scores good accuracy was found. Most patients with CIDP revealed hypoechoic enlarged nerves (Class 1). BUS/NUP was useful to identify CIDP (sensitivity>70%), while the UPSS showed high sensitivity and positive/negative predictive values (N/PPV) in the diagnosis of CIDP (>85%). Immune-mediated neuropathies mostly show regional nerve enlargement. The HS was suitable to identify CMT patients with an HS C5 points. |
| 29 | 2017 | Niu | 9 CMT1A *vs* 28 CIDP *vs* 14 HC | CSA of a total of ten predetermined sites for median and ulnar nerves were measured. | The CSA values in CMT1A were higher at all sites, with a gradual and homogeneous enlargement from  distal to proximal. The CSA values in CIDP were higher than in HC at all sites and three different types of nerve morphology was observed: little enlargement (CIDP type 1), mild nerve enlargement (CIDP type 2) and segmentally prominent enlargement (CIDP type 3). |
| 30 | 2018 | Harting | 80 CIDP (35 untreated and 45 treated) | UPSS score was computed.  Homogeneity of the nerves maximum fascicle size was measured at the level of the upper arm (for median and ulnar nerve) and the knee (for fibular nerve). Echogenicity was also evaluated. | Mean UPSS did not differ significantly between untreated and treated patients. Conversely, a different distribution of ultrasound patterns between the two groups: significant nerve enlargement (UPSS ≥ 5) with predominantly hypoechoic fascicle pattern and without prominent perifascicular tissue increase was the predominant pattern in untreated patients (class 1), whereas in the treated group was predominant significantly enlarged nerves (UPSS≥ 5) and an increased echointensity of the fascicles or the perifascicular tissue at > 50% of all measurement points (class 2). |
| 31 | 2018 | Pitarokoili | 108 nerve segments of 18 CIDP | Evaluate the individual role of HRUS and MRI in assessing the morphological alterations of all peripheral nerves and  plexus in CIDP and investigate the  significance of CSA enlargement through correlations with MRN markers of nerve integrity and oedema.  HRUS protocol: CSA of median, ulnar, radial, tibial, fibular, sural nerves and brachial plexus. | The maximal CSA-HRUS values correlated significantly with maximal CSA-MRI for all nerves measured. However, neuroimaging of the  proximal segments of the tibial nerve and the  lumbrosacral plexus requires MRI measurements. |
| 32 | 2018 | Tan | 9 Diabetic demyelinating sensorimotor neuropathy *vs* 10 CIDP | CSA of median, ulnar, superficial radial, tibial, peroneal and sural nerves | CIDP patients had markedly larger nerves at  the proximal and non-entrapment sites of the upper limbs. |
| 33 | 2019a | Fisse | 20 CIDP (stable *vs* progressive) | Echogenicity of median, ulnar, radial, tibial and fibular nerves  was measured, according to Padua classification in three category hypoechogenic, mixed, hyperechogenic. | Patients of the stable group exhibited more frequently hypoechoic arm nerves, while hyperechoic arm nerves were observed more often in patients of the progressive group. Differences were not significant for the leg nerves. Echogenicity of the arm nerves in CIDP may be used as a prognostic marker, but not as a follow-up tool for evaluating clinical changes. |
| 34 | 2019b | Fisse | 20 CIDP (stable *vs* progressive) | Ultrasound was performed every 6 months. Intranerve CSA variability was computed in median, ulnar, radial, tibial, fibular and sural nerves. BUS were also calculated. | The intranerve cross-sectional-area (CSA) variability of the nerves of the lower extremity increased with disease progression, whereas it remained unchanged in patients with a stable or remitting disease course. |
| 35 | 2019 | Goedee | 6 patients with clinical CIDP phenotype without electrodiagnostic features of demyelination | CSA, fascicle size, echogenicity, and screening for increased nerve  vascularization of median nerves and brachial plexus trunks. | Nerve ultrasound may also be helpful in identifying the more elusive patients in which electrodiagnostic features of demyelination are absent, helping to reduce the cost of IVIg trials. |
| 36 | 2019 | Grimm | 33 GBS *vs* 34 CIDP (50% with acute-onset) | UPSS score was evaluated. Homogeneity of the nerves was evaluated for the median, the ulnar, and the tibial nerve. The maximum fascicle size was measured for median, ulnar and fibular nerves. Lastly, echointensity of CIDP patients was assessed according to Padua classification. | Enlargement (UPSS, UPSA and UPSC and not UPSB) was significantly higher in CIDP compared with GBS.  In most patients with GBS, fascicles were not or only regionally restricted enlarged, whereas in CIDP, a significantly more heterogeneous distribution of fascicle pattern was found ranging from no enlargement to diffuse enlargement affecting most fascicles measured. Increased echointensity of the nerves further arises only in CIDP. After 6 months, in CIDP, the significant nerve enlargement persisted, whereas in GBS, all segments almost normalized.  Enlarged roots/vagus in combination with ultrasonic sensory sparing pattern might facilitate differentiation of GBS and CIDP in the early stage. |
| 37 | 2019 | Niu | 54 CIDP (21 patients follow-up of more 6 months) | Relationship between CSA and electrophysiological characteristics and how nerve morphology changes over time in CIDP after standard treatment. | No correlation was observed between the maximum CSA and motor conduction velocity. There were segmental nerve enlargements at 61% of sites with conduction block or temporal dispersion. Among patients with clinical improvement after immunotherapy, CSA decreased to normal in 5, increased in 10, and were unchanged in 4. |
| 38 | 2019 | Pitarokoili | 17 CIDP (10 worsening vs 7 stable) | CSA of median, ulnar, radial, tibial, fibular and sural nerves. BUS was calculated for each patient and each visit. | BUS and a maximum BUS of 4 points at study initiation identified patients with disease progression (sensitivity 80%, specificity 88%). BUS seem to represent early markers for clinical progression in CIDP. |
| 39 | 2019 | Puma | 11 CIDP | Comparison between high frequency ultrasound (HFUS 18–20 MHz) *vs* Ultra-high frequency ultrasound (UHFUS 30–70 MHz). Nerve and fascicle CSA, vascularization, and echogenicity of median and ulnar nerves, bilaterally. were assessed. | UHFUS allowed for a more precise estimation of  fascicle size and number than the HFUS. We were able to identify nerve vascularization in 4/11 patients at UHFUS only. UHFUS gives more detailed information on the changes in the internal nerve structure in CIDP patients. |
| 40 | 2020 | Crump | 50 cases (21 CIDP and 22 other neuropathies) |  | CIDP were more likely to have multiple sites of enlargement, as well as more pronounced nerve enlargement. The presence of any moderately enlarged nerve segment predicted definite CIDP with sensitivity of 81% and specificity 77%. |
| 41 | 2020 | Gamber | 20 CIDP *vs* 79 HC | Semiautomated evaluation of echogenicity, fascicle count and fascicle cross-sectional area (CSA) of median, ulnar, radial, tibial and fibular nerves. | Semiautomated evaluation of echogenicity, fascicle count, and fascicle CSA is reliable. Clinically progressive CIDP patients had a lower echogenicity than healthy controls and stable CIDP patients, suggesting that this parameter is useful for detecting clinically progressive CIDP patients and should be used in clinical context or intraindividual course. |
| 42 | 2020a | Herraets | 100 chronic immune neuropathies (CIN), including CIDP, MADSAM and MMN | Short sonographic protocol (median nerve at forearm, upper arm, and C5 nerve  root) to determine its diagnostic accuracy respect the EFNS/PNS criteria of CIDP/MMN. | Sensitivity and specificity of the short sonographic protocol for CIN were 87.4% and 67.3%, respectively. With addition of nerve ultrasound 44 diagnoses of CIN were established compared to 33 diagnoses with NCS alone. |
| 43 | 2020b | Herraets | 100 chronic immune neuropathies (CIN), including CIDP, MADSAM and MMN | Determine diagnostic accuracy of ultrasound respect the EFNS/PNS 2010 criteria of CIDP/MMN and determine the added value in the detection of treatment-responsive patients. | Sensitivity and specificity of nerve ultrasound were 97.4% and 69.4%, while of NCS were 78.9% and 93.5%. The added value of nerve ultrasound in detection of treatment-responsive chronic inflammatory neuropathy was 21.1% compared to NCS alone. |
| 44 | 2020 | Niu | 43 CIDP *vs* 8 CMT1A *vs* 105 HC | Exploring the role of vagus nerve in discriminating CIDP and CMT1A. CSA of the vagus, median and ulnar nerves were measured at 10 sites. | CIDP patients showed higher vagus nerve CSA than HC, and lower respect CMT1A. High diagnostic accuracy of study of vagus nerve was found for the diagnosis of CIDP and CMT1A. For CIDP, the area under the curve (AUC) was 0.866; a cutoff value of 1.5 mm^2^ yielded a sensitivity of 79.3% and a specificity of 91.3%. Moreover, vagus nerve CSA was positively correlated with the maximum CSA of median/ulnar nerve and with the alteration in mean median/ulnar nerve CSA in CIDP during follow-up. |
| 45 | 2021 | Athanasopoulos | 203 CIDP patients (group A: 182 with EFNS CIDP criteria *vs* Group B: 21 with no EFNS-CIDP criteria) | HRUS, CSA of the median, ulnar, and radial, tibial, fibular and sural nerves. The brachial plexus was also assessed. BUS was computed. | Group B showed typical morphological changes in nerve ultrasound. |
| 46 | 2021 | Dorner | 10 CIDP *vs* 3 POEMS | UPSS and homogeneity score was assessed. Moreover, echointensity was also studied. | CIDP patients showed greater CSA enlargement and higher UPSS, UPSA and HS compared with POEMS, although POEMS patients exhibited enlarged nerves exceeding reference values (not restricted to entrapment sites). CIDP patients visualized both increased and decreased echointensity, while POEMS syndrome patients pictured hypoechoic nerves with hyperechoic intraneural connective tissue. |
| 47 | 2021 | Du | 18 ATTRv *vs* 13 CIDP *vs* 14 HC | CSA of median, ulnar, sciatic, tibial, common peroneal, and sural nerves. CSA variability was also evaluated. | CIDP patients showed a higher CSA respect HC and ATTRv. CSA variability of median nerves in CIDP groups was significantly higher than ATTRv and HC, reaching the AUC of 0.8 with high sensitivity (0.692) and specificity (0.833). |
| 48 | 2021 | Fionda | 23 CIDP (3-year follow-up) | To provide information on the evolution of  the pathological load.  CSA of median, ulnar and peroneal nerve. | Peripheral nerve size tends to increase over time in patients with CIDP (in particular in median nerve), in correlation with clinical and alectrophysiological changes, in particular in those nerve segments that undergo a predominantly demyelinating damage. |
| 49 | 2021 | Niu | 104 CIDP *vs* 26 CMT1A *vs* 111 HC | CSA of upper limb (median, ulnar) and lower limb nerves (tibial, fibular, sural). Either median or ulnar nerve is enlarged, the patient has nerve enlargement of upper limbs. Either tibial or fibular nerve is enlarged, the patient has nerve enlargement of lower limbs | 86% CIDP patients had upper limb nerve enlargement, while only 67% had lower limb nerve enlargement. In CIDP patients with normal upper limb ultrasound, 56% (5/9) would have lower limb nerve enlargement. All CMT1A patients had both upper and lower limb nerve enlargement. Addition of lower limb nerve ultrasound showed no added value in diagnosis of CMT1A but could be supplementary for CIDP when upper limb ultrasound is normal. |
| 50 | 2021 | Telleman | 126 CIDP (52 typical, 74 atypical) *vs* 72 MMN *vs* 35 CIAP | To explore the natural course of nerve size development and determine its prognostic value. CSA of median, ulnar and the brachial plexus. | No significant correlations between nerve size and clinical outcome measures were found in CIDP. No predictive effects of sonographic nerve size were found in CIDP group. |
| 51 | 2022 | Brünger | 95 CIDP *vs* 82 NIAP (no inflammatory axonal polyneuropathy) | Definition of adjusted BUS (aBUS) characterized by the CSA measurement of median nerve at the forearm and at the upper arm, ulnar nerve at the forearm and at the upper arm, radial nerve at spiral groove and sural nerve between the gastrocnemius muscle. Each enlargement at different sites was scored as 1 point, for a total values from 0 to 6. | CSA of patients with CIDP was enlarged at all six nerve sites compared with NIAP patients, but the enlargement of the sural nerve and the left ulnar nerve on the upper arm was not statistically significant. The cut-off of ≥2 points in the aBUS to diagnose a CIDP reached a specificity of 95% and a sensitivity of 36%. Applying the aBUS cut-off at those patients classified as “possible” or “probable” CIDP, a specificity of 94% and a sensitivity of 59% was reached. |
| 52 | 2022 | Erdmann | 20 CIDP *vs* 19 Critical Illness Neuropathy (CIP) *vs* 27 Chemotherapy-Induced Neuropathy (CIN) | Compare nerve echogenicity in different primarily axonal or demyelinating polyneuropathies by the evaluation of median, ulnar, radial, fibular and tibial nerves. | The nerves of patients with progressive CIDP significantly differed from the hyperechogenic nerves of patients with other polyneuropathies at the upper limb nerves. The echogenicity of patients with stable CIDP, CIP and CIN did not show much difference. |
| 53 | 2022 | Leonardi | 25 CIDP *vs* 11 ATTRv | CSA of brachial plexus, median, ulnar, peroneal nerves for a total of 26 sites. | Nerve ultrasound showed a lower number of affected nerve segments a in patients with ATTRv than in patients with CIDP. Nerve CSA in patients with ATTRv was significantly lower than in CIDP patients. In the two ATTRv patients with electrodiagnostic study data compatible with a CIDP diagnosis, data were comparable to those in patients with CIDP. |
| 54 | 2022a | Niu | 120 CIDP and 34 POEMS | CSA were measured on the bilateral median nerve, ulnar nerve and brachial plexus. Moreover, homogeneity of CSA along the nerve was considered. | CSAs at all sites were larger in patients with CIDP and POEMS syndrome than in healthy controls.  The ratio of maximum/minimum CSA of the median nerve was significantly larger in CIDP than in POEMS syndrome. Nerve CSA enlargement was more homogeneous along the same nerve in individual POEMS patients. The addition of nerve ultrasound to nerve conduction studies significantly improves the differential diagnosis between the two diseases. |
| 55 | 2022b | Niu | 18 therapy-naive CIDP and 45 followed up CIDP | Explore whether nerve ultrasound and its changing trend could predict the response to immune treatment in CIDP.  CSA was measured at ten sites on both the median and ulnar nerves. | The response rate to steroids (95%) was significantly higher than that to IVIG (70%) in patients with normal or moderately enlarged CSA, while there was no significant difference in the response rate between steroid therapy (84%) and IVIG (75%) in patients with markedly enlarged CSA. CSAs decreased in 15 patients during follow-up, most of whom had good IVIG and steroid responses (83%) and no need for immune suppressant treatment (82%)  Patients with normal or moderately enlarged CSA may respond better to steroids than to IVIG. The decrease in CSA after treatment may also indicate better prognosis. |
| 56 | 2022 | Oka | 6 anti-MAG neuropathy *vs* 10 typical CIDP (tCIDP) *vs* 5 MADSAM *vs* 17 HC | CSA of C5–C7 cervical nerve roots, median and ulnar nerves. Inter- and intra-nerve variability was also calculated. To quantify the degree of nerve enlargement at entrapment sites compared to non-entrapment sites, the ratio of the CSA at the entrapment site and the CSA at the nearest proximal non-entrapment site was computed. | Cervical nerve root CSA was significantly larger at every examined site on both sides in anti-MAG neuropathy than in MADSAM and HCs but were comparable to those in t-CIDP. Peripheral nerve enlargements were greatest at common entrapment sites (ie, wrist and elbow) in anti-MAG neuropathy, a pattern shared with t-CIDP but not with MADSAM.  The degree of nerve enlargement at entrapment sites compared to non-entrapment sites was significantly higher in anti-MAG neuropathy than in t-CIDP. |
| 57 | 2023 | Hashiba | 26 CIDP vs 34 POEMS | To investigate US-based radiomic analysis. CSA and echogenicity of the median and ulnar nerves were evaluated. | The CSA were more enlarged in patients with CIDP although without significant differences. Nerve echogenicity was significantly more heterogeneous in patients with CIDP. The radiomic analysis yielded four features with the highest AUC value of 0.83. The machine-learning model showed an AUC of 0.90. |
| 58 | 2023 | Hildebrand | 11 ALS *vs* 5 CIDP *vs* 5 CMT *vs* 15 controls | CSA and nerve microvascular blood flow of tibial nerve were evaluated. Microvascular blood flow was scored as following: grade 0= no blood flow; grade 1= 1 or 2 focal color-encoded spots; grade 2= 1 linear color-encoded line or >2 focal color-encoded spots; grade 3= >1 linear color-encoded line. | CSA was significantly larger in CMT and CIDP compared to ALS and controls, and microvascular blood flow was higher in the CIDP cohort. |
| 59 | 2023 | Kitaoji | 39 CMT *vs* 19 CIDP | CSA of median and ulnar nerves, C6 roots were examined. | CMT patients showed larger nerve CSA at all measurement points than CIDP patients. AUC values of nerve CSA at each measurement point ranged from 0.70 to 0.81. |
| 60 | 2023 | Tan | 34 CIDP, 15 AIDP, 16 axonal neuropathies, 30 HC | Utility of ultrasound pattern sub-score A (UPSA) and intra- and inter-nerve cross-sectional area (CSA) variability in the diagnostic evaluation of demyelinating neuropathies | Significantly enlarged nerve CSA was observed in CIDP and AIDP with significantly higher UPSA in CIDP compared to the other groups. By using a cut-off UPSA score ≥7, the performance of UPSA in differentiating CIDP from other neuropathies including AIDP was excellent (AUC = 0.943) with high sensitivity (89.3%), specificity (85.2%) and positive predictive value (73.5%). No significant differences in intra- and inter-nerve CSA variability between the three groups. |
| 61 | 2023 | Yoshikawa | 39 patients (14 typical CIDP, 7 MADSAM CIDP, 4 DADS and 14 MMN) | CSA of median and ulnar nerve, C5, C6, and C7 nerve roots at their exit point.  The Wrist-Forearm Index (WFI= wrist CSA/forearm CSA), Elbow-Upper arm Index (EUI= elbow CSA/upper arm CSA), Intra-nerve CSA Variability (INCV= Maximal CSA/minimal CSA) were calculated within the same nerve. | Significant differences were observed among typical CIDP, multifocal CIDP, distal CIDP, and MMN in CSA at the forearm and upper arm in the median nerves. Patients with multifocal CIDP had  lower WFI and EUI and higher INCV than the other groups. The multifocal CIDP group had more pronounced nerve enlargement at the forearm  and upper arm, especially in the median nerve, and the WFI and EUI were lower. |
| 62 | 2023 | Yun | 25 CIDP (19 typical, 6 atypical) | CSA, echogenicity, and vascularity of the bilateral median and ulnar nerves. | Focal nerve enlargement was found in at least one segment in all subjects, with more pronounced enlargement in typical CIDP. A focused ultrasound study (involving only the median and ulnar nerves) is sensitive for the detection of nerve enlargement in CIDP. |
| 63 | 2024 | Naito | 12 CMT1A *vs* 17 CIDP | CSA of median, ulnar, C5, C6, tibial, fibular and sural nerve for a total of 38 sites. Enlargement site number (ESN), the number of sites/levels that exhibited nerve enlargement, was also evaluated. | The screening ESNs in the intermediate region and lower extremities were greater in patients with CMT1 than in patients with CIDP and greater than the ESN in the distal region. The ESNs in the intermediate region and lower extremities significantly differed among patients with typical CIDP, CIDP variants, and CMT1. The combined upper and lower extremity ESNs exhibited the highest AUC (0.92). |
| 64 | 2024 | Niu | 135 CIDP (99 typical, 10 MADSAM, 15 DADS, 9 pure motor, 2 pure sensory) | CSA median, ulnar and brachial plexus were measured bilaterally in all subjects.  The nerve enlargement pattern in each patient was classified according to the amount of enlargement in the proximal, intermediate, and distal segments. | Moderately increased or normal CSA was found in 61% typical CIDP and 78% pure motor CIDP patients, while 67% DADS and 70% MADSAM patients had significantly increased CSA. A diffuse enlargement was seen in 51% typical CIDP, 50% MADSAM, 25% DADS and 33% pure motor CIDP patients, while a proximal regional enlargement pattern in 12% typical CIDP, 10% MADSAM, 50% DADS and 44% pure motor CIDP patients. Patients with diffusely moderate enlargement patterns and those with proximal regional enlargement showed a higher response rate to glucocorticoids than to IVIg. |
| 65 | 2024 | Puma | 28 patients (12 CIDP, 6 distal CIDP and 10 anti‑MAG) *vs* 10 HC | UHF-US study (median and ulnar nerves bilaterally) in order to distinguish between different dysimmune neuropathies, though the analysis of the nerve and fascicle CSA. | UHF‑US was reliable in differentiating immune neuropathies from controls when using mean and/or segmental nerve and/or fascicle CSA; furthermore, fascicle ratio (fascicle/nerve CSA) was a reliable factor for differentiating d‑CIDP from other types of polyneuropathies. The fascicle CSA appears to be more increased in CIDP and its variant than in anti‑MAG neuropathy. UHF‑US offers information beyond simple nerve CSA and allows for a better characterization of the different forms of dysimmune  neuropathies. |

AIDP= Acute Inflammatory Demyelinating Polyneuropathy; ALS= Amyotrophic Lateral Sclerosis; ATTRv= TTR Amyloidosis, variant; AUC= Area Under the Curve; BUS= Bochum ultrasound score; CIDP= Chronic Inflammatory Demyelinating Polyneuropathy; CIN= Chemotherapy-Induced Neuropathy; CIP= Critical Illness Neuropathy; CMT= Charcot-Marie-Tooth disease; CSA= Cross-Sectional Area; CSF= Cerebrospinal Fluid; DADS= Distal Acquired Demyelinating Symmetric neuropathy; GBS= Guillain-Barré Syndrome; HC= Healthy Controls; INCAT= Inflammatory Neuropathy Cause and Treatment; MADSAM= Multifocal acquired demyelinating sensory and motor neuropathy; MAG= Myelin Associated Glycoprotein; MGUS= Monoclonal gammopathy of undetermined significance; MMN= Multifocal Motor Neuropathy; MRC= Medical Research Council; NIAP= no inflammatory axonal polyneuropathy; NPV= negative predictive value; NSI= Nerve Size Index; NUP= neuropathy ultrasound protocol; POEMS= Polyneuropathy, Organomegaly, Endocrinopathy, Monoclonal gammopathy, Skin changes; PPV= positive predictive value; UHFUS= Ultra High Frequency ultrasound; UPSS= Ultrasound Pattern Sum Score; US= ultrasound.
