# Supplementary material for: Role of Imaging in Chronic Inflammatory Demyelinating Polyneuropathy: A Systematic Review
Source: Eur J Neurol. 2025 Jun 1;32(6):e70226. doi: 10.1111/ene.70226 (PMC12127570; doi:10.1111/ene.70226)
Supplement: Supplementary file 2 — Table S2. Summary of nerve MRI research articles included in the systematic review. [file ENE-32-e70226-s002.docx]

**Supplementary Table 2: Summary of nerve MRI research articles included in the systematic review**

| Reference | Years | Article | Study population | Aim and MRI outcome | Main findings |
| --- | --- | --- | --- | --- | --- |
| 71 | 1996 | Schady | 3 CIDP | Not defined | Enlargement of lumbar and cervical root whereas the thoracic region was unaffected. |
| 72 | 1997 | Kubawara | 10 CIDP | T2W, TIW, T1W after gadolinium. Axial imaging of the arm or forearm performed with 1.5-tesla MRI  In patients with focal conduction abnormalities MRI was used to image the demyelinate focus in the median or ulnar nerve.  In patients without focal conduction abnormalities mid-forearm was imaged. | 8 patients showing demyelinate features median or ulnar nerve trunk. These demyelinate foci showed nerve enlargement with high signal intensity on T1W images.  In 4 patients with progressive illness or relapse, the enlarged segment showed gadolinium enhancement that disappeared during remission induced by immune therapies. The other 4 were in the steady phase and showed no gadolinium enhancement of the enlarged nerves.  The 2 patients who showed conduction slowing, but no focal demyelinate focus, had neither nerve enlargement nor gadolinium enhancement. |
| 73 | 1998 | Mizuno | 3 CIDP | Not defined | Enlargement of peripheral nerve trunks and nerve roots.  Nerve trunks and nerve roots showed markedly high signal intensity in the T2-W images, with no or minimal gadolinium enhancement in the peripheral nerve trunks and marked enhancement in the nerve roots. |
| 74 | 1999 | Duggins | 14 CIDP | BP and LPS coronal and axial fast spin echo T2-W and coronal T1-W images.  1.5 Tesla MRI system was used for 10 patients and a 0.5 Tesla system for 4 cases. | 8 patients display Hypertrophy of cervical roots and BP six of whom also had hypertrophy of the LSP.  Of 11 patients who received gadolinium, 5/6 cases with hypertrophy and 1/5 without hypertrophy demonstrated enhancement.  All patients with hypertrophy had a relapsing–remitting course and a significantly longer disease duration. |
| 75 | 1999 | Midroni | 16 CIDP vs 13 DC (5 CMT1A, 2 MND, 1 Diabetes, 1 SCA, 1 brachial plexopathy, canal stenosis, transverse myelitis) | **Methods:** T1-W sagittal and axial views before and after administration of gadolinium. | 11/16 CIDP displayed enhancement of cauda equina while none of 13 DC.  Nerve roots were enlarged, most significantly in the extraforaminal region, in 3 CIDP, and in one CMT. |
| 76 | 2010 | Adachi | 13 CIDP *vs* 11 HC | **Aim:** clarify the MR characteristics of the BP and LSP in CIDP  **Methods:** STIR, T1W, T1W post gadolinium and DWI. | 16/24 CIDP display diffuse enlargement  and abnormally high signals on STIR.  A slightly high signal was detected in 12/24 plexuses on T1W, and a high-intensity signal was detected in 10/18 plexuses on DWIs with high ADC values.  Contrast enhancement was in 6/19 plexuses and mild in all cases. |
| 77 | 2011 | Sinclair | 10 CMT1A *vs* 9 CIDP *vs* 10 HC | **Aim:** evaluation of CSA of sciatic nerve.  **Methods**: One mid-thigh was imaged on STIR and CSA evaluated. | CSA of sciatic nerve area was enlarged in both CMT1A and CIDP compared with controls and in CMT1A compared with CIDP (p<0.001).  Median areas were 67.6 mm^2 for CIDP group, 135.9 mm^2 for the CMT1A group and 43.3 mm^2 for the control group. |
| 78 | 2012 | Beydoun | 3 CIDP | **Aim:** to evaluate MRI abnormalities in 3 CIDP  **Methods:** T2-W and T1-W with and without gadolinium on the neuraxis (brain, cervical spine, thoracic spine, and lumbosacral regions). | Nerve hypertrophy and marked enlargement of peripheral nerves, trunks, and roots with nerve root enhancement and minimal enhancement of the trunks. |
| 79 | 2013 | Tanaka | 15 CIDP *vs* 30 HC | **Aim:** to evaluate usefulness cervical nerve roots MRI for diagnosis of CIDP  **Methods:** C5–C8 root diameters determined on coronal STIR images. Signal intensities of these roots were quantified as nerve-to-muscle contrast-to-noise ratios (CNRs). | The root diameters displayed no significant differences between groups. The nerve-to-muscle CNRs were significantly higher in the patients with CIDP. |
| 80 | 2014 | Rajabally | 5 Multifocal-CIDP | **Methods:** BP STIR | All patients displayed marked and bilateral hyperintensity on T2 STIR |
| 81 | 2014 | Shibuya | 33 CIDP (27 typical and 6 Multifocal-CIDP) | **Aim:** to evaluate distribution and patterns of nerve hypertrophy in CIDP.  **Methods:** MR neurography with 3D reconstruction of STIR images. | Nerve enlargement detected in 88% of the patients. Typical CIDP patients displayed symmetric and  root-dominant hypertrophy, whereas Multifocal-CIDP patients displayed multifocal fusiform hypertrophy  in the nerve trunks. |
| 19 | 2015 | Pitarokoili | 9 CIDP | CSA of the lower limb nerves (tibial, fibular and sural nerves) through US and MRI evaluation. | Patients with higher disability showed isolated enlarged fascicles and increased CSA of the peripheral nerves, whereas two of them showed atrophic fascicles. Nerve ultrasound and MRI findings show the same morphological fascicle alterations in peripheral nerves in correlation to ODSS. |
| 82 | 2015 | Tomura | 9 CIDP *vs* 4 BP palsy of unknown origin *vs* 2 MND *vs* 1 Cervical Spondylosis *vs* 2 muscle atrophy of the upper extremity with unknown origin | **Aim & Methods:** To compare T2-W fast spin-echo IDEAL (T2W IDEAL-FSE) with STIR to determine which sequence is superior to image the BP. | Qualitatively, each score for T2W IDEAL-FSE was significantly higher than that for STIR. |
| 83 | 2016 | Lozeron | 33 CIDP | **Aim**: to evaluate plexus MRI value in the diagnosis of CIDP with an atypical presentation.  **Methods:** Plexus MRI performed on the most affected territory (brachial or lumbar). Were assessed: plexus trophicity, T2-STIR signal intensity and gadolinium enhancement. | 11 CIDP patients with initial atypical clinical presentation had abnormal plexus MRI. Hypertrophy of the proximal plexus and/or extraforaminal roots was found in 8 cases and Gadolinium enhancement in 2 cases. Abnormalities were more frequent on brachial (86%) than lumbosacral MRI (29%) and asymmetrical (72%) |
| 84 | 2016 | Markvardsen | 14 CIDP treated with SCIg *vs* 14 DC | **Aim:** to evaluate if DTI correlates to muscle strength or impairment.  **Methods:** MRI of sciatic and tibial nerves obtaining T2- and DTI-W with fat saturation.  DTI, FA and ADC were calculated. | FA was lower in CIDP (0.38 ± 0.07 versus 0.45 ± 0.05)  ADC was higher in CIDP (1735 ± 232 versus 1593 ± 116 × 10−6 mm2).  FA in the sciatic nerve distinguishes CIDP from controls with a sensitivity and a specificity of 92.9%. |
| 27 | 2017 | Goedee | 23 CIDP *vs* 28 MMN | **Aim:** To compare the diagnostic performance of US and MRI applied to the BP in CIDP and MMN.  **Methods:** T1-W spin-echo, STIR, Sagittal-T2W spin-echo, T1W post Gadolinium. | MRI displayed enlargement and/or a pathological T2-hyperintense signal of the BP in 17/23 (74%) CIDP and 14/28 (50%) MMN.  Enlargement without T2-hyperintense signal was found in 2/28 (7%) patients with MMN, but in none of the patients with CIDP, and T2-hyperintense signal without enlargement in 7/23 (30%) CIDP and 5/28 (18%) MMN.  No gadolinium enhancement in any of the patients.  The diagnostic performance of neuroimaging ranged from 61–73% to 83%. |
| 85 | 2017a | Hiwatashi | 14 CIDP *vs* 9 HC | **Aim:** To evaluate the usefulness of 3D nerve-sheath signal increased with inked rest-tissue rapid acquisition of relaxation enhancement imaging (SHINKEI) in patients with CIDP.  **Methods:** signal-to-noise ratio (SNR), contrast ratio (CR), and the size of the cervical ganglions and roots were measured. | SNRs of the ganglions and roots were larger in CIDP than HC. CRs of the ganglions and roots were larger in CIDP than HC. Sizes of the ganglions and the roots were larger in CIDP than HC. |
| 86 | 2017b | Hiwatashi | 21 CIDP *vs* 15 non-CIDP | **Aim**: evaluate whether 3D SHINKEI in the LSP could identify CIDP patients  **Methods**: The SNR, contrast-to-noise ratio (CNR), contrast ratio (CR) and the size of the lumbar ganglions and roots were measured. | The SNRs, CNRs and CRs were larger in patients with CIDP. ROC analysis showed the best diagnostic performance with the CNR of the roots. |
| 87 | 2017 | Ishikawa | 13 CIDP *vs* HC | **Aim:** to visualize peripheral nerves in  CIDP and compare results with HC.  **Methods:** Whole-body MRI neurography based on DWIBS was performed. Peripheral nerve volumes were calculated from serial axial MRI images. | BP and LSP and nerve root were visualized with 3D reconstruction. Volumes ranged from 8.7 to 49.5 cm³/m² in the BP and nerve roots and from 10.2 to 53.5 cm³/m² in the LSP and nerve roots. CIDP had significantly larger volumes than controls and volume was positively correlated with disease duration. |
| 88 | 2017 | Jongbloed | 18 typical CIDP *vs* 40 MMN *vs* 9 Multifocal-CIDP | **Aim:** to evaluate correlation between distribution of BP MRI abnormalities and clinical weakness, and to evaluate the value of BP MRI in predicting disease course and response to treatment in MMN, Multifocal-CIDP and typical CIDP.  **Methods:** bilateral T2-W STIR of BP. | BP MRI abnormalities were detected in 45% of patients.  Abnormal MRI did not predict disease course in terms of patterns of weakness, sensory disturbances or response to treatment.  Asymmetrical clinical syndromes (MMN and Multifocal-CIDP) were associated with asymmetrical radiological abnormalities, whereas symmetrical abnormalities predominated in typical CIDP. |
| 89 | 2017 | Kronlage | 18 CIDP vs 18 HC | **Aim:** assess diagnostic accuracy of DTI in CIDP  **Methods:** MR neurography of upper and lower extremity nerves (median, ulnar, radial, sciatic, tibial) performed by single-shot DTI sequences at 3.0 T. Nerve fractional anisotropy (FA), mean diffusivity (MD), RD, and AD were obtained | Nerve FA was decreased to a mean of 0.42 ± 0.08 in CIDP patients compared with 0.52 ± 0.04 in HC (P < 0.001). This decrease in FA was a result of an increase of RD (P = 0.02), whereas AD did not differ between groups. parameters, FA showed best diagnostic accuracy. Optimal cutoff for an average FA of all analyzed nerves was 0.47, yielding a sensitivity of 0.83 and a specificity of 0.94. |
| 90 | 2017 | Lichtenstein | 11 CIDP *vs* 11 HC | **Aim:** to evaluate utility of nerve DTI, nerve CSA, and muscle MRI multiecho Dixon for assessing proximal nerve injury in CIDP.  **Methods:** multiparametric MRI protocol with DTI of the sciatic nerve and assessment of muscle proton-density fat fraction of the biceps femoris and the quadriceps femoris muscles by multiecho Dixon MRI.  Patients were longitudinally evaluated by MRI, clinical examination, and nerve conduction studies at baseline and after 6 months. | In sciatic nerves of CIDP, mean CSA was significantly higher and FA value was significantly lower than HC.  Muscle proton-density fat fraction was significantly higher in thigh muscles of CIDP than HC.  MRI parameters showed high reproducibility at baseline and 6 months.  Advanced MRI parameters demonstrate subclinical proximal nerve damage and intramuscular fat accumulation in CIDP. |
| 91 | 2018 | Hiwatashi | 10 CIDP *vs* 5 HC | **Aim**: to evaluate usefulness of simultaneous T2 mapping and neurography with SHINKEI in LSP to distinguish CIDP from HC.  **Methods**: T2 relaxation time and size of DRG and nerves of LSP at L3-S1. | T2 relaxation time of DRG and the nerves of LSP were longer in CIDP compared to HC. Size of nerves was larger in CIDP. No significant difference for size of DRG between groups. |
| 31 | 2018 | Pitarokoili | 18 CIDP (108 nerve segments) | **Aim:** to assess correlation nerve HRUS and MRI  **Methods:** median, ulnar, radial, tibial and fibular nerve and lumbar and cervical plexus were examined with HRUS and MRN.  MRI: fat-saturated T2-W 3D sequence of the LSP, and a fat-  saturated T2-W turbo spin-echo (TSE) sequence for visualization of nerve morphology. A single-shot spin-echo  echo planar imaging (EPI) DTI sequence was acquired at the mid-thigh, the lower leg and the upper arm of one randomly selected side. | CSA values correlated for all nerves between US and MRN. CSA in HRUS correlated with the nerve T2-W signal increase as well as with DTI parameters such as FA. HRUS-CSA of the interscalene BP correlated significantly with the MRN-CSA and nT2 signal of the L5 and S1 roots of the LSP. |
| 92 | 2019 | Fargeot | 7 probable CIDP *vs* 13 possible CIDP *vs* 18 no criteria for CIDP *vs* 10 definite CIDP | **Aim:** to evaluate usefulness of plexus MRI in the diagnosis of CIDP without definite EFNS/PNS electrodiagnostic criteria is currently unclear.  **Methods:** MRI of the LSP, the BP or both. Sequences: 3D T1-W, 3D STIR pre and post gadolinium, Coronal and axial (LSP) or coronal and sagittal (BP) post-gadolinium T1-W. | Plexus MRI displayed abnormalities in 22/38 (58%) patients including increased nerve signal intensity on T2-W images in 22/22 (100%), nerve enlargement in 20/22 (91%) and contrast enhancement in 8/22 (36%). Plexus MRI enabled the expert committee’s final diagnosis to be adjusted in 7/38 (18%) patients. In conjunction with nerve conduction studies was a supportive criterion to classify 7/24 (29%) patients as definite CIDP. MRI abnormalities were more asymmetrical and less diffuse in not definite-CIDP than in definite-CIDP-D. |
| 93 | 2019 | Hiwatashi | 13 CIDP vs 5 HC | **Aim:** to evaluate the usefulness of simultaneous apparent T2 mapping and neurography with SHINKEI to distinguish CIDP from HC.  **Methods**: T2 relaxation time and the size of the cervical ganglia and roots of BP were measured. | T2 relaxation times of the ganglia and roots were longer in CIDP. The sizes of the ganglia and the roots were larger in CIDP. |
| 94 | 2019 | Thammongkolchai | 9 CIDP | **Methods:** MRI of lumbar spine (T1-W, T1-W post gadolinium, T2-2) | 8 patients exhibited gadolinium enhancement and thickening of multiple spinal nerve roots  and/or cranial nerves. |
| 95 | 2019 | Felizas | 10 CIDP *vs* 10 HC | **Aim:** to evaluate the role of MR micro-neurography to detect morphological and relaxometric changes in distal tibial nerves in CIDP and their associations with clinical and electrophysiological features.  **Methods:** multiple MR parameters, including the number of fascicles (N), fascicles diameter (FD), total fascicles area (FA), epineurium area (EA), total nerve area (NA), fascicles to nerve ratio (FNR) and quantitative T2 and proton density (PD) were investigated on high resolution MR images of the distal tibial nerve. | Median NA and FA were significantly increased in the CIDP population. No correlation between the parameters investigated and clinical or electrophysiologic features. |
| 96 | 2020 | Feng | 45 CIDP (32 typical CIDP *vs* 6 CIDP-DADS *vs* 5 Multifocal CIDP *vs* 1 motor CIDP). | **Aim:** to prospectively evaluate the discrimination of typical CIDP from its variants according to electrophysiology and magnetic resonance neurography (MRN).  **Methods:** measurement of CSA at C7-C8 and L4-S1 nerve roots. | CSAs of C7, C8, L4, L5, and S1 in typical CIDP patients were significantly thicker than in DADS and Multifocal-CIDP. No difference in terms of CSA between DADS and Multifocal-CIDP. |
| 97 | 2020 | Jomier | 28 suspected CIDP | **Aim**: determine the additional  benefit of plexus MRI in patients referred to tertiary centers with baseline clinical and electrophysiological characteristics suggestive of typical or atypical CIDP.  **Methods:** According to the prevailing symptoms, patients underwent either lumbosacral and/or BP MRI, thus providing T2-STIR images in the coronal plane. | MRI was abnormal in 5/37 patients (14%). |
| 98 | 2020 | Oudeman | 13 CIDP *vs* 10 MMN *vs* 12 SMA *vs* 30 HC | **Aim:** to assess and compare diagnostic performance of qualitative and (semi-) quantitative MRI and ultrasound for distinguishing CIDP and MMN from segmental SMA**.**  **Methods:** MRI of the BP, using STIR neurography and DTI sequences. | Roots and trunks with severe hypertrophy were only seen in CIDP on both STIR and MRN.  Increased signal intensity was found in MMN  (0%–33%), SMA (0%–10%), and CIDP (15%–58%) on STIR. |
| 99 | 2020 | Shah | 10 CIDP *vs* 10 HC | **Aim:** to quantify diameter and CSA of lumbosacral nerve roots and explore imaging characteristics of sciatic nerves, in CIDP versus HC using MRI**.**  **Methods:**  MRI of the LSP and both thighs (Sciatic nerve) was performed. A volumetric coronal T1-(MPRAGE) sequence of the LSP and Axial 3D STIR for sciatic nerves were acquired. | Lumbosacral nerve root diameter and CSA were significantly increased in CIDP compared to HC  (mean diameter 6.0 ± 1.1 mm vs 4.8 ± 0.3 mm; p = 0.006), with a high sensitivity (89 %) and specificity (90%).  Sciatic nerve CSA was significantly increased in CIDP with qualitative MRI changes (hyperintensity of several degree in CIDP). |
| 100 | 2020 | Su | 31 CIDP *vs* 21 HC | **Aim:** to evaluate characteristics of nerve abnormalities in CIDP using MR neurography and to examine the diagnostic efficiency.  **Methods:** T1/T2-W turbo spin-echo sequences and T1-W post gadolinium were performed for neurography of the brachial and LSP and cauda equina. | Vagus (n = 11), trigeminal (n = 12), and intercostal nerves (n = 10) were hypertrophic. 19/31 (61.3%) CIDP displayed BP hypertrophy. 25/31 (80.6%) CIDP displayed LSP hypertrophy.  Patterns of hypertrophy included uniform hypertrophy (17 [54.8%] BP and 21 [67.7%] LSP), and multifocal fusiform hypertrophy (2 [6.5%] BP and 4 [12.9%] LSP) was present. Enlarged and/or contrast-enhanced cauda equina was found in 3 (9.7%) and 13 (41.9%) CIDP. Diameters of the BP and LSP were significantly larger in CIDP than HC. The largest AUC was obtained for the L5 nerve. |
| 101 | 2020 | Van Rosmalen | 19 CIDP *vs* 17 MMN *vs* 14 HC | **Aim**: to assess interrater variability between radiologists by using a predefined scoring system that allowed the distinction of no, possible, or definite nerve thickening. | Raters agreed in 26 of 50 (52%) BP images, evidencing that interrater reliability of qualitative evaluation of BP MRI is low. |
| 102 | 2020 | Wu | 21 CIDP *vs* 21 HC | **Aim:** to determine the value of morphological features of lumbosacral nerve roots on MRN in diagnosing CIDP and analyse correlations with electrophysiological parameters.  **Methods:** CSA and signal intensities (SI) of L3 to S1 nerve roots were measured. | CIDP displayed significantly increased CSA and SI from L3 to S1 nerve roots.  CSAs of L5 or S1 nerve root correlated positively with central latency and negatively with conduction velocity of tibial nerve. |
| 103 | 2021 | Benoit | 30 focal CIDP  (18 plexus neuropathy F-PN, 7 senori/sensorymotor F-SMN, 5 purely motor F-PM) | **Aim & Methods:** 26/30 underwent LSP and/or BP MRI, according to symptoms location.  Sequences: STIR, T1-W post Gadolinium | -18 F-PN patients underwent brachial or LSP MRI. All had abnormalities of the symptomatic plexus and roots (hypertrophy and/or increased STIR signal intensity)  - 4/7 F-SMN underwent BP MRI: 1 revealed multifocal fusiform enlargement of the right BP with proximal contralateral root abnormalities, and 1 revealed increased signal intensity and thickening of the proximal ulnar nerve.  - 3/5 F-PM three had diffuse hypertrophy and increased signal intensity of the LSP. |
| 104 | 2021 | Su | 37 CIDP *vs* 37 HC | **Aim:** to evaluate diagnostic performance and abnormalities of plexus via quantitative multisequence MRN for CIDP**.**  **Methods:** nerve diameter, nerve-to-muscle T2 signal intensity ratio (nT2), contrast-enhanced ratio (CR), fractional anisotropy (FA), and apparent diffusion coefficient (ADC) were determined in both plexus, and tractographies were performed. | The sizes of nerve roots were larger in CIDP. CR, nT2, and ADC were significantly higher, while FA was lower in CIDP than HC. FA had the highest sensitivity (0.809) and area under the curve (AUC) (0.925), while the highest specificity was 0.961 for CR in single parameters.  The combination of FA and CR has the highest sensitivity, specificity, accuracy, and AUC in the LS plexus. FA had a negative correlation with the duration in the CIDP. |
| 105 | 2021a | van Rosmalen | 47 CIDP *vs* 29 MMN *vs* 40 MND vs 10 HC | **Aim:** to systematically study nerve architecture of the BP in patients with CIDP, MMN, MND and HC using quantitative MRI techniques.  **Methods**: MRI of BP with diffusion parameters, T2 relaxation times and fat fraction. | FA was lower in CIDP than HC, MND and MMN.  RD was higher in CIDP than HC, MND and MMN.  T2 relaxation time was elevated in CIDP compared to MND. Fat fraction was lower in CIDP and MMN compared to MND. |
| 106 | 2021b | van Rosmalen | 50 CIDP *vs* 31 MMN *vs* 42 DC | **Aim:** to develop a quantitative approach to assess abnormalities on MRI of the BP and the cervical roots in CIDP and MMN and to evaluate interrater reliability and its diagnostic value.  **Methods:** systematically measured cervical nerve root sizes on MRI bilaterally (C5, C6, C7) in the coronal and sagittal planes next to the ganglion and 1 cm distal from the ganglion | Nerve root size was larger in patients with CIDP and MMN compared to controls at all predetermined anatomical sites. |
| 107 | 2022 | Beecher | 9 BP (5 Multifocal CIDP *vs* 4 MMN) and 6 LSP (4 Multifocal CIDP *vs* 2 MMN) | **Aim:** to evaluate if plexus MRI can distinguish Multifocal CIDP from MMN among pathologically (nerve biopsy) confirmed cases.  **Methods:** brachial and LSP MRI studies including orthogonal  T1-W pre- and post‐gadolinium, T2-W fat suppression or STIR were reviewed. | MRI hypertrophy occurred solely in mutlifocal CIDP (89%, 8/9) with T2-hyperintensity in both. |
| 108 | 2022 | Thirouin | 75 possible CIDP (EFNS/PNS 2021) | **Aim:** to evaluate the value of diagnostic tests to support the diagnosis of CIDP in patients with possible CIDP and to identify prognostic factors of therapeutic success.  **Methods:** T1-W, T2-W, T1-W post gadolinium, fat-suppressed sequences. | MRI did not predict therapeutic response  5/32 (16%) displayed Abnormal MRI.  MRIs were considered consistent with CIDP when increased signal intensity on T2-W, hypertrophy, and/or gadolinium enhancement was identified in the cauda equina, the lumbosacral or the cervical nerve roots, or the brachial or LSP. |
| 109 | 2022 | Van Rosmalen | 40 CIDP *vs* 27 MMN *vs* 34 MND | **AIM:** to evaluate if intra-spinal roots are also affected. This MRI study systematically visualized intra-spinal nerve roots, i.e., the ventral and dorsal roots, in CIDP, MMN and MND**.**  **Methods:**  MRI scan of the cervical intra-spinal roots. Systematically measured intra-spinal nerve root sizes bilaterally in the transversal plane at C5, C6 and C7 level. | Patients with MMN and CIDP with a motor phenotype had thicker ventral roots compared to patients with CIDP with a sensorimotor phenotype, while patients with CIDP with a sensory phenotype had thicker dorsal roots compared to patients with a sensorimotor phenotype and with MND. |
| 110 | 2022a | Wu | 20 CIDP *vs* 10 axonal acquired polyneuropathies (APN) vs 20 HC | **Methods**: 3D T2-W fat-suppressed  and DTI sequences of the LSP. Parameters analysed: CSA, FA, ADC of L3-S1 roots. | CSA and ADC were higher in CIDP than APN and HC  FA was reduced in both CIDP and  APN compared to HC but there was no difference in the two groups.  To differentiate CIDP from axonal polyneuropathies, CSA had better diagnostic accuracy with cut-off value of 29.46 mm2 and sensitivity and specificity of 75% and 100% respectively. |
| 111 | 2022b | Wu | 18 CIDP *vs* 18 age and sex-matched HC | **Aim**: detect the diffusion characteristics of lumbosacral nerve roots and explore their correlations with electrophysiological parameters of lower extremity nerves.  **Methods:** DTI of LSP was performed in all subjects and FA, AD, RD and MD of lumbosacral nerve roots were measured | CIDP patients showed significantly lower FA as well as higher AD, RD, and MD values of lumbosacral nerve roots. FA had the best diagnostic accuracy with an area under the curve of 0.914 and optimal cut-off value of 0.27.  FA and RD may serve as potential markers reflecting the conduction function of tibial and common peroneal nerves. |
| 112 | 2023 | Preisner | 12 CIDP *vs* 12 HC | **Aim:** to evaluate changes in nerves trough MRI in CIDP vs HC along six years of follow-up  **Methods:** T2-W, DTI, MTI and T2 relaxometry, DTI.  Baseline MRI:2016  Follow-up MRI: 2022 | Over time, nerve CSA decreased in CIDP patients, especially at the lumbosacral plexus. Longitudinally, changes in CSA correlated with changes in clinical scale: high initial nerve CSA was inversely correlated with changes in the INCAT/ODSS. FA correlated with electrodiagnostic testing both cross-sectionally and longitudinally. |
| 113 | 2023 | Su | 51 CIDP | **Aim:** to evaluate relationships between multisequence MR neurography findings, electrophysiological parameters, and clinical characteristics in CIDP.  **Methods:** Multiparameter-MRN includes diameter, nerve-to-muscle T2 signal intensity ratio (nT2), CR, FA and ADC of bilateral plexus nerve roots. | Moderate correlations were found between motor nerve conduction velocity and distal motor latency in nerve diameter, nT2, FA, and ADC, respectively. The correlations between CR and sensory nerve conduction velocity and peak latency were moderate, and ADC had a positive correlation with compound motor action. |

AD= axial diffusivity; ADC= Apparent Diffusion Coefficient; BP= Brachial plexus; IDP= Chronic Inflammatory Demyelinating Polyneuropathy; CMT= Charcot-Marie-Tooth disease; CNR= contrast-to-noise ratios; CR= contrast ratio; CSA= Cross-Sectional Area; DADS= Distal Acquired Demyelinating Symmetric neuropathy; DC= disease controls; DRG= Dorsal Root Ganglia; DTI= Diffusion tensor imaging; DWI= Diffusion Weighted Imaging; DWIBS= DTI whole-body imaging with background body signal suppression; EPI= echo planar imaging; FA= fractional anisotropy; HC= Healthy Controls; LSP= lumbosacral plexus; MD= mean diffusivity; MMN= Multifocal Motor Neuropathy; MND= Motor Neuron Disease; MRI= Magnetic Resonance Imaging; MRN= magnetic resonance neurography; PD= proton density; RD= radial diffusivity; SCA= Spinocerebellar Ataxia; SCIg= Subcutaneous Immunoglobulin; SHINKEI= nerve-sheath signal increased by inked rest-tissue rapid acquisition with relaxation enhancement imaging; SMA= spinal muscular atrophy; STIR= Short Time Inversion Recovery; T2W= T2 weighted; TIW= T1 weighted; TSE= fat-saturated T2-W turbo spin-echo; US= Ultrasound
